# Comments on the manuscript "Hepatorenal syndrome: an update"

**DOI:** 10.1590/S1516-31802008000100013

**Published:** 2008-01-03

**Authors:** Charles Soper

I read the article "Hepatorenal syndrome: an update"^1^ in São Paulo Med J with great interest. When I published a small study in 1996 with favorable results from the use of relatively small doses of BQ123 in severely affected patients, I tried unsuccessfully to attract commercial interest to pursue the investigation. The physiological effects of ET-1 in healthy volunteers in Rabelink's study^2^ seemed to affect them similarly to features seen in early hepatorenal syndrome (HRS) at comparable plasma levels, irrespective of the important question of intrarenal generation. Such effects in healthy subjects were completely abolished by selective ET-A blockade^3^. The use of combined ET-A and B antagonists has resulted in hypotension, but no other additional renal haemodynamic effects.^4^.

I have recently become aware of the paper by Moore et al. reporting a lack of benefit from a combined ET blocker in HRS,^5^ and interestingly also reporting hypotension, but as yet selective ET-A blockade does not seem to have been reattempted therapeutically. Is the author aware of any such investigation in process?
