# Supplementary material for: Comments on the manuscript "Hepatorenal syndrome: an update"
Source: Sao Paulo Med J. 2008 Jan 3;126(1):67. doi: 10.1590/S1516-31802008000100013 (PMC11020517; doi:10.1590/S1516-31802008000100013)
Supplement: Related texts

# Reply: Response

**Authors:** Tércio Genzini

Thank you for your comments.

Unfortunately, we have no clinical experience with endothelin antagonists. However, we have been treating many patients with hepatorenal syndrome (HRS) and we are very interested in any kind of better alternatives for them than albumin plus terlipressin, transjugular intrahepatic portosystemic shunt (TIPS) and others, while waiting for liver transplantation.
